# Diagnostic role of circulating MiR-21 in colorectal cancer: a update meta-analysis

**DOI:** 10.1080/07853890.2020.1828617

**Published:** 2020-10-14

**Authors:** Tong Liu, Duo Liu, Shangwei Guan, Mei Dong

**Affiliations:** Pharmaceutical Department, Harbin Medical University Cancer Hospital, Harbin, China

**Keywords:** MicroRNA-21, colorectal cancer, diagnosis, meta-analysis

## Abstract

**Aim:**

MicroRNA-21 is an oncogenic miRNA that modulates the expression of multiple cancer-related target genes. We conducted this meta-analysis to assess diagnostic role of circulating miR-21 in CRC, hoping to choose the best biomarker in CRC diagnosis.

**Methods:**

We searched the PubMed, CNKI and WanFang database to identify records related to diagnostic role of circulating miR-21 in CRC. The search words were “microRNA-21”, “miRNA-21”, “colorectal cancer”, “colorectal carcinoma” and “diagnosis”. The searched articles were published before 14^th^ July 2020.

**Results:**

We got 18 studies to conduct this meta-analysis including 1129 blood specimens of CRC patients and 951 control specimens. The meta-analysis showed that the pooled sensitivity and specificity of circulating miR-21 for CRC diagnosis were 77% (95% CI, 70–82) and 83% (95% CI, 78–88). The combined positive likelihood ratio (PLR) was 4.20 (95% CI, 3.12–5.66) and the combined negative likelihood ratio(NLR) was 0.30 (95% CI, 0.23–0.38). The diagnostic odds ratio (DOR) was 16.48 (95% CI 10.09–26.91) and the area under the summary receiver operating characteristic curve (SROC) for the included studies was 0.87(95%CI, 0.84–0.90).

**Conclusion:**

Our meta-analysis results suggest that circulating miR-21 has a potential diagnostic value with moderate sensitivity and good specificity for CRC.

## 1. Introduction

Colorectal cancer (CRC) is a major cause of cancer-related death worldwide [1]. Despite prevention and screening, this disease has an increasing incidence and high mortality. It is the most frequently occurring malignancy in the word, accounting for more than one million cases and 500,000 deaths per year [2,3]. Several CRC screening tests, including faecal occult-blood test and colonoscopy, are frequently used in the detection of CRC. Nevertheless, none of these tests have been established as well-accepted screening tools due to their invasiveness, high cost, or low sensitivity [4]. Therefore, the search for a more sensitive, easy to be detected and representative biomarker is of great significance for CRC early diagnosis and monitoring.

MicroRNAs (miRNAs) are non-coding RNA molecules of about 20 nucleotides long that are involved in regulation of gene expression at the transcriptional and post-translational levels. MiRNAs are extensively implicated in many complex physiological processes such as cellular differentiation, proliferation, and apoptosis [5]. Numerous studies indicate that miRNAs can play an important role as reliable biomarkers for cancer detection and prognostic prediction, and even as novel targets for cancer therapy [6,7].

MiR-21 is an oncogenic miRNA that modulates the expression of multiple cancer-related target genes such as PTEN, TPM1, and PDCD and has been shown to be overexpressed in various human tumors [8–10]. Elevated expression of miR-21 in the tumour tissues of CRC has shown to be as an independent prognostic and predictive biomarker [11–14]. Therefore, there will be great significance to explore diagnostic role of circulating miR-21 in CRC. Although several studies have reported the diagnostic effect of miR-21 in blood samples of CRC patients, the conclusion is various and the number of included patients of each study is not enough [15,16]. Hence, we conducted this meta-analysis to assess diagnostic role of circulating miR-21 in CRC, hoping to choose the best biomarker in CRC diagnosis.

## 2. Materials and methods

This meta-analysis was performed following the guidelines of the preferred reporting items for systematic reviews and meta-analysis (PRISMA) statement [17].

### 2.1. Search strategy

We searched the PubMed, China National Knowledge infrastructure (CNKI) and WanFang database to identify records related to diagnostic role of circulating miR-21 in CRC. The search words were “microRNA-21”, “miRNA-21”, “colorectal cancer”, “colorectal carcinoma” and “diagnosis”. The searched articles were published before 14^th^ July 2020.

### 2.2. Inclusion criteria

(1) Patients were diagnosed with colorectal cancer. (2) MiRNA-21 expressions in blood specimens of CRC patients were detected by real time quantitative RT-PCR assay. (3) The included study at least reported sensitivity and specificity of miR-21 in diagnosis of CRC patients.

### 2.3. Exclusion criteria

(1) Letters, reviews, conference abstracts, animal experiments, fundamental research, and duplicated studies were excluded. (2) Studies that did not estimate the diagnostic role of miR-21 in CRC were excluded. (3) Studies whose data could not be used for meta-analysis were excluded.

### 2.4. Data extraction

According to inclusion and exclusion criteria, two reviewers independently screened all studies to extract data. Extracted data included the first author’s name, publication year, Country, sample size, source of miR-21, cancer type, true positive(TP), false positive(FP), false negative(FN), true negative(TN), sensitivity and specificity. Disagreements were resolved by the third-party adjudication.

### 2.5. Quality assessment

The quality of each included study was assessed by Quality Assessment of Diagnostic Accuracy Studies(QUADAS) tool.

### 2.6. Statistical analysis

STATA15.1 software was used to perform the statistical analysis for this meta-analysis. The sensitivity and specifificity of each study were used to construct a 2 × 2 contingency table. The bivariate meta-analysis model was employed to summarise the sensitivity, specifificity, positive likelihood ratio(PLR), negative likelihood ratio (NLR) and diagnostic odds ratio (DOR) and generate the bivariate summary receiver operating characteristic curve. The pooled sensitivity, specificity and other related indexes across studies were calculated using a random-effects model. Heterogeneity between studies was evaluated using the chi-squared and I^2^ tests. A probability value of *p* < .05 was taken as being statistically significant and I^2^≥50% indicated the existence of significant heterogeneity [15]. The presence of publication bias was analysed by Deeks' funnel plot asymmetry test. Statistical significance was considered, if publication bias was present at *p* < .1 [16].

## 3. Results

### 3.1. Literature search and study characteristics

**Figure 1.**
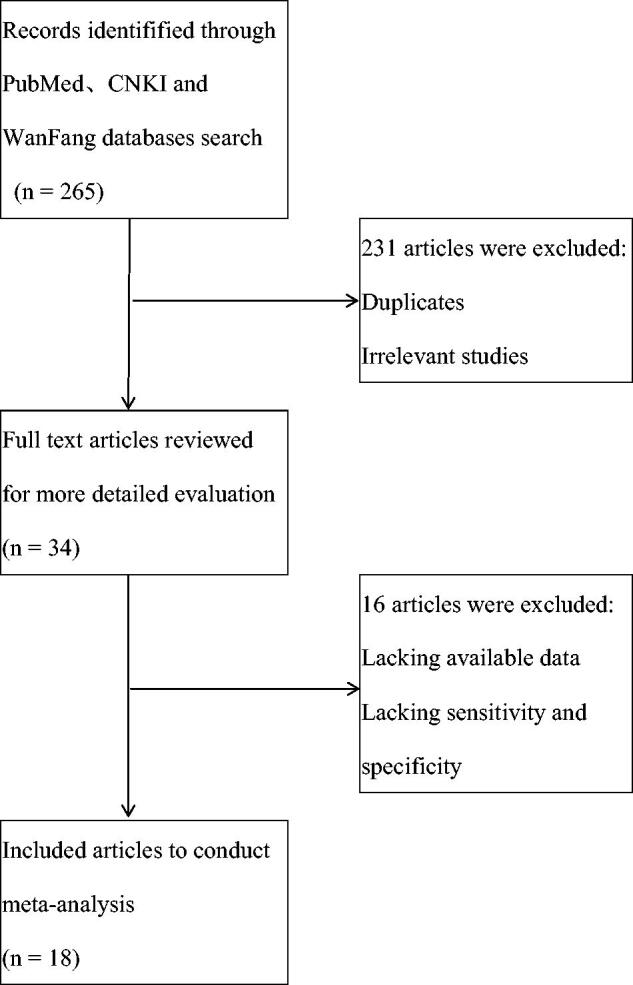
Study flow diagram.

As shown in Figure 1, we identified 265 records from PubMed, CNKI and WanFang Databases. After reading titles and abstracts to exclude the duplicates and irrelevant studies, 34 studies remained to review full text to extract available data. As some data in studies could not be used, finally we got 18 studies to conduct meta-analysis to evaluate the diagnostic role of circulating miR-21 in CRC [18–35].

The characteristics of included studies are shown in Table 1. We got 1129 blood specimens of CRC patients and 951 control specimens. The control specimens were from the age and sex matched healthy people. The publication year of included studies were from 2012 to 2020 and the 18 studies came from 8 different countries(China, Slovak, Russia, Japan, Egypt, America, Iran, Germany). Based on QUADAS scores, all included studies were evaluated as high quality.

**Table 1.** Characteristics of included studies in this meta-analysis.

Study (year)	Age (year)		Gender (M/F)		Country	Source of control	Source of miR-21	Samples size		Cancer type	Test method	Reference gene	Diagnosis parameters						QUADAS scores
	P	C	P	C				P	C				TP	FP	FN	TN	Sen	Spe	
Sazanov et al. [18] (2017)	NA		NA		Russia	Healthy volunteers	Plasma	31	34	CRC II-IV	qRT-PCR (SYBR Green)	U6 snRNA	20	5	11	29	0.65	0.85	12
								Saliva	34			34		33	3	1	31	0.97		0.91
Basati et al. [19] (2014)	55.35 ± 10.13	55.00 ± 10.35	21/19	22/18	Iran	Healthy volunteers	Serum	40	40	CRC	qRT-PCR (SYBR Green)	RNU6B	31	9	9	31	0.77	0.78	13	
Liu et al. [20] (2013)	NA		NA		China	Healthy volunteers	Serum	200	80	CRC	qRT-PCR (TaqMan)	miR-16	130	12	70	68	0.65	0.85	12	
Fouad et al. [21] (2017)	49.54 ± 15.37	49.18 ± 16.17	26/24	25/25	Egypt	Healthy volunteers	Serum	50	50	CRC	qRT-PCR(TaqMan)	RNU6B	41	22	9	28	0.82	0.56	12	
Sarlinova et al. [22] (2016)	40-86	44-86	46/25	58/22	Slovak	Healthy volunteers	Serum	71	80	CRC	qRT-PCR (TaqMan)	RNU48	51	26	20	54	0.72	0.68	12	
Bastaminejad et al. [23] (2017)	NA		21/19	21/19	Iran	Healthy control	Serum	40	40	CRC	qRT-PCR (SYBR Green)	miR-16	34	11	6	29	0.86	0.73	12	
							Stool	40	40				34	8	6	32	0.86	0.81		
Jin et al. [24] (2020)	50.9 ± 9.5	42.32 ± 9.23	38/42	28/22	China	Healthy control	Serum	80	50	CRC	qRT-PCR (ABI7500)	U6 snRNA	73	7	7	43	0.91	0.86	13	
Toiyama et al. [25] (2013)	NA		NA		Japan	Healthy volunteers	Serum	186	53	CRC	qRT-PCR (TaqMan)	cel-miR-39	154	5	32	48	0.83	0.91	12	
Sabry et al. [26] (2018)	51.97 ± 12.18	48.47 ± 15.16	19/16	52/49	Egypt	Healthy control	Serum	35	101	CRC	qRT-PCR (SYBR Green)	RNU6B	32	5	3	96	0.91	0.95	13	
Hua et al. [27] (2018)	35-86	32-69	30/22	25/20	China	Healthy volunteers	Serum	52	45	CRC	qRT-PCR (SYBR Green)	U6 snRNA	44	9	8	36	0.85	0.80	13	
Chen et al. [28] (2019)	58.0 ± 16.5	56.5 ± 16.0	39/33	15/15	China	Healthy control	Plasma	72	30	CRC	qRT-PCR	NA	50	1	22	29	0.69	0.97	12	
Song et al. [29] (2013)	59.18 ± 11.1	55.44 ± 12.1	30/10	40/16	China	Healthy control	Serum	40	56	CRC	qRT-PCR(TaqMan)	miR-16	30	17	10	39	0.75	0.69	13	
Wang et al. [30] (2012)	63	46	17/15	9/30	China	Healthy individuals	Serum	32	39	CRC	qRT-PCR (SYBR Green)	miR-16	28	10	4	29	0.88	0.74	12	
Ogata-Kawata et al. [31] (2014)	35-65	35-65	55/33	3/8	Japan	Physical screening individuals	Serum	88	11	CRC	qRT-PCR (TaqMan)	miR-451	54	1	34	10	0.61	0.91	13	
Zhang et al. [32] (2014)	NA		NA		China	Normal control	Serum	41	30	CRC	qRT-PCR	U6 snRNA	21	6	20	24	0.51	0.79	11	
Du et al. [33] (2014)	61.1 ± 12.7	61.7 ± 12.6	30/19	30/19	China	Healthy control	Plasma	49	49	CRC	NA	NA	37	3	12	46	0.76	0.93	11	
Luo et al. [34] (2013)	67.1 ± 11.0	61.7 ± 6.4	25/25	25/25	Germany	Healthy control	Plasma	80	144	CRC	qRT-PCR (TaqMan)	miR-16	42	27	38	117	0.52	0.81	13	
Kanaan et al. [35] (2012)	60 ± 11	61 ± 9	7/13	7/13	America	Healthy control	Plasma	30	30	CRC	qRT-PCR (TaqMan)	U6 snRNA	27	3	3	27	0.9	0.9	13	

M: Male; F: Female; P: Patient; C: Control; Sen: Sensitivity; Spe: Specificity; NA: Not Available.

### 3.2. Data analysis

**Figure 2.**
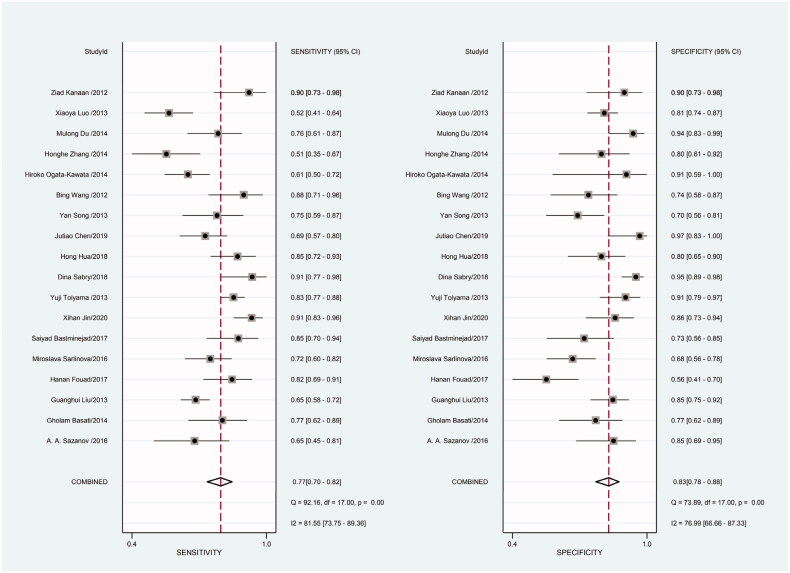
Forest plot of included studies assessing the sensitivity and specificity of circulating miR-21 in CRC.

Heterogeneity in sensitivity and specificity was detected in the 18 studies (I^2^=81.55% and I^2^=76.99%, respectively), suggesting significant heterogeneity in sensitivity and specificity (Figure 2). For this reason the random-effects model was employed. The meta-analysis showed that the pooled sensitivity and specificity of circulating miR-21 for CRC diagnosis were 77% (95% CI, 70–82) and 83% (95% CI, 78–88), respectively.

**Figure 3.**
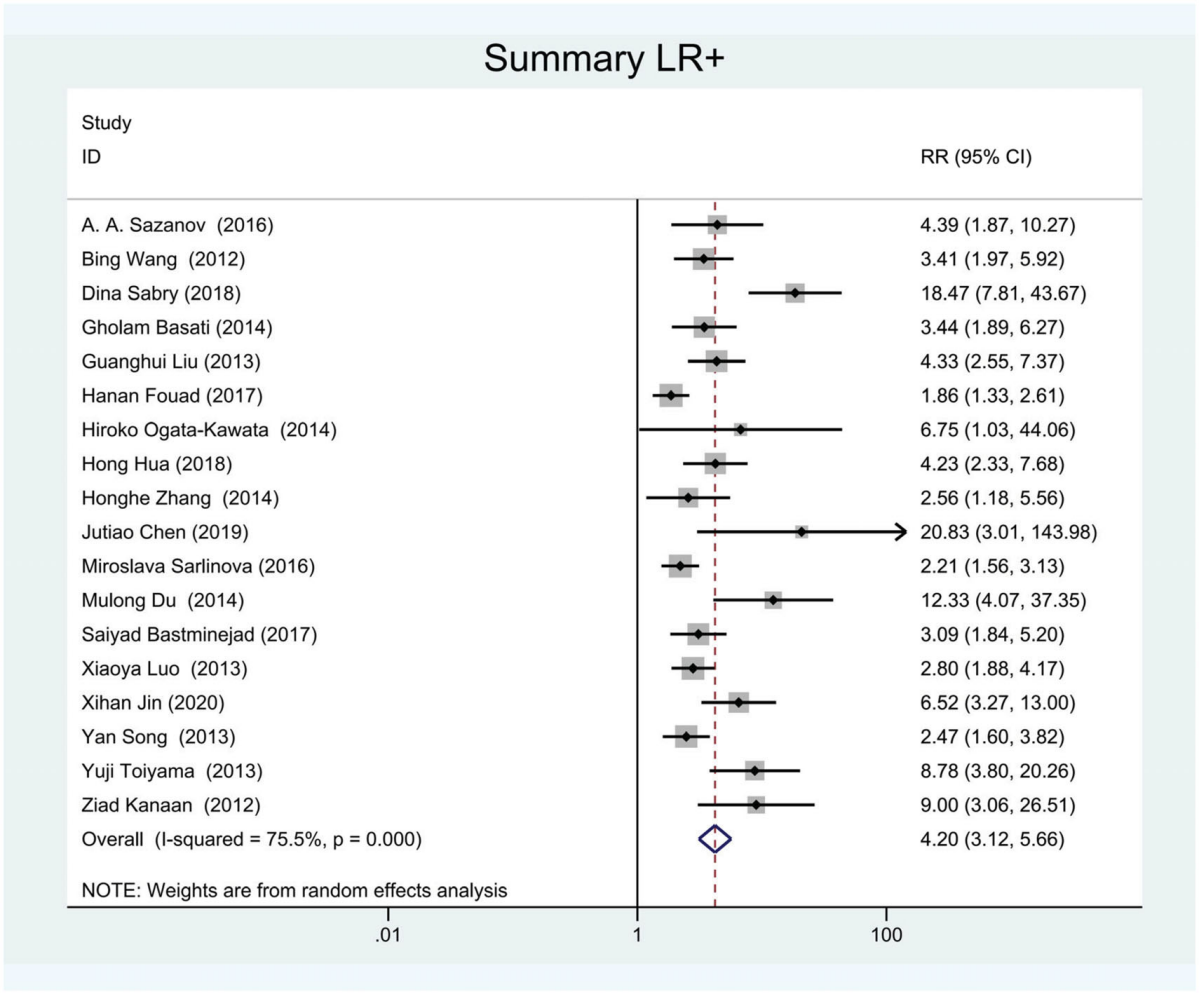
Forest plot of included studies assessing the PLR of circulating miR-21 in CRC.

**Figure 4.**
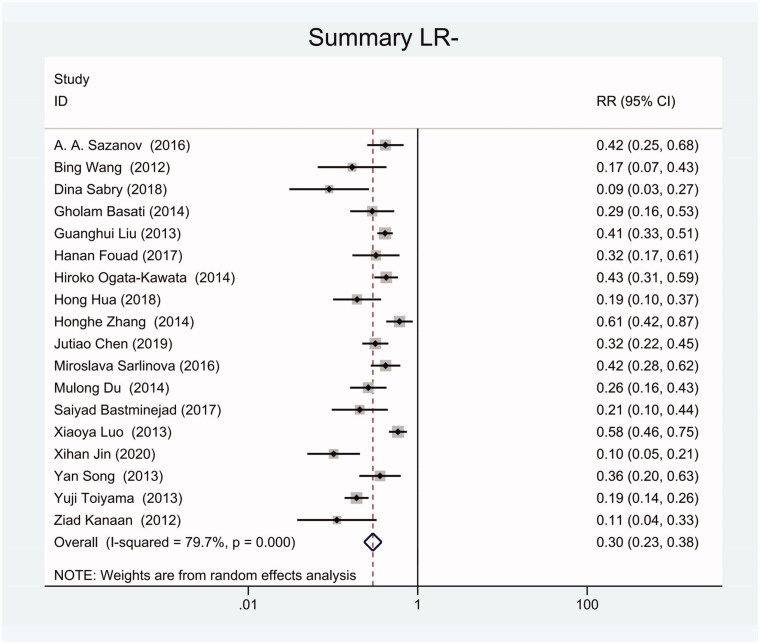
Forest plot of included studies assessing the NLR of circulating miR-21 in CRC.

As the positive likelihood ratio(PLR) and negative likelihood ratio(NLR) have been considered more clinically valuable compared to the specificity and sensitivity. In this meta-analysis, the combined PLR was 4.20 (95% CI, 3.12–5.66) (Figure 3), which indicated that patients with CRC had a nearly four-fold greater chance of having an elevated miR-21 in blood specimens compared with healthy individuals. The combined NLR was 0.30 (95% CI, 0.23–0.38) (Figure 4).

**Figure 5.**
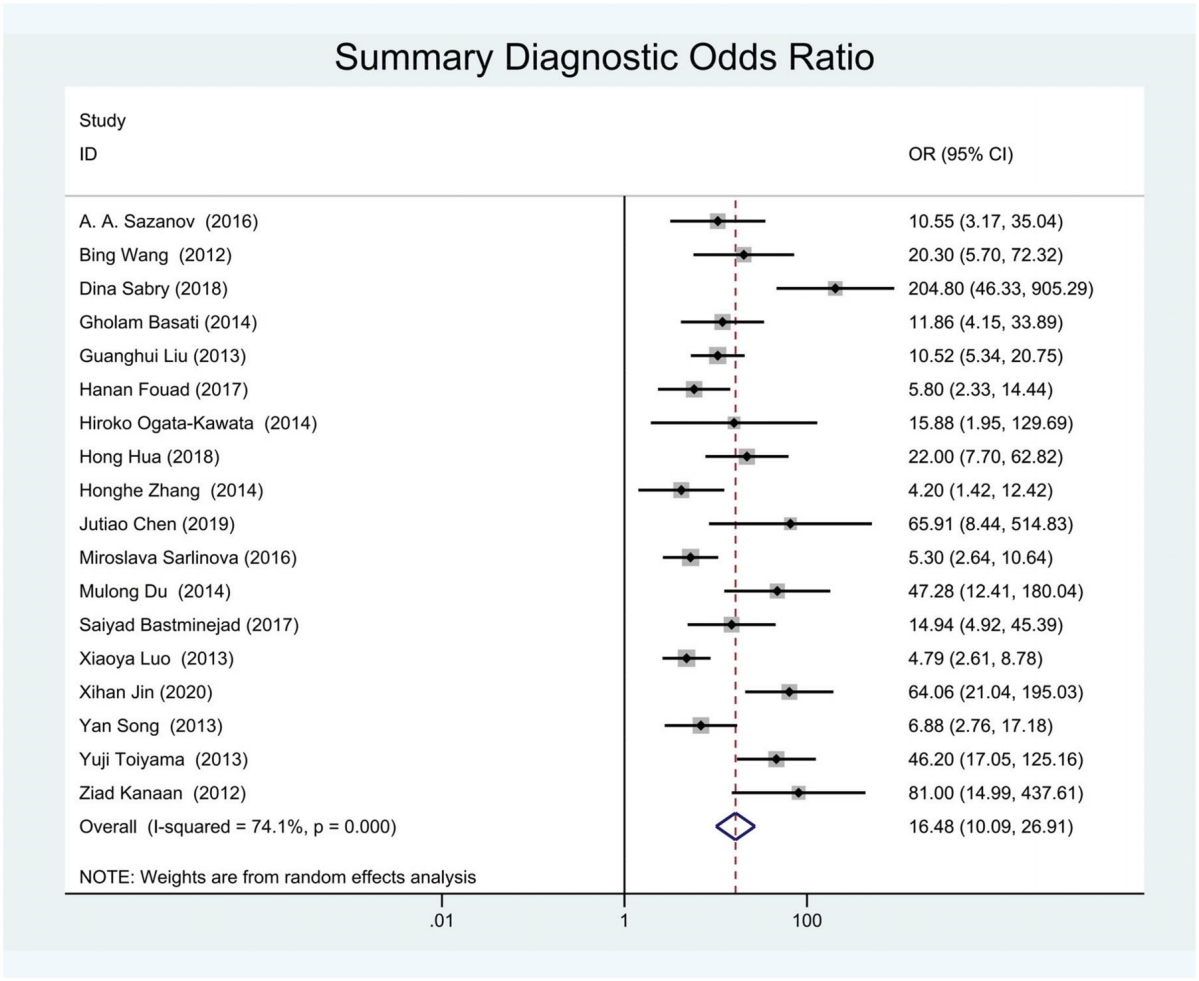
Forest plot of included studies assessing the DOR of circulating miR-21 in CRC.

**Figure 6.**
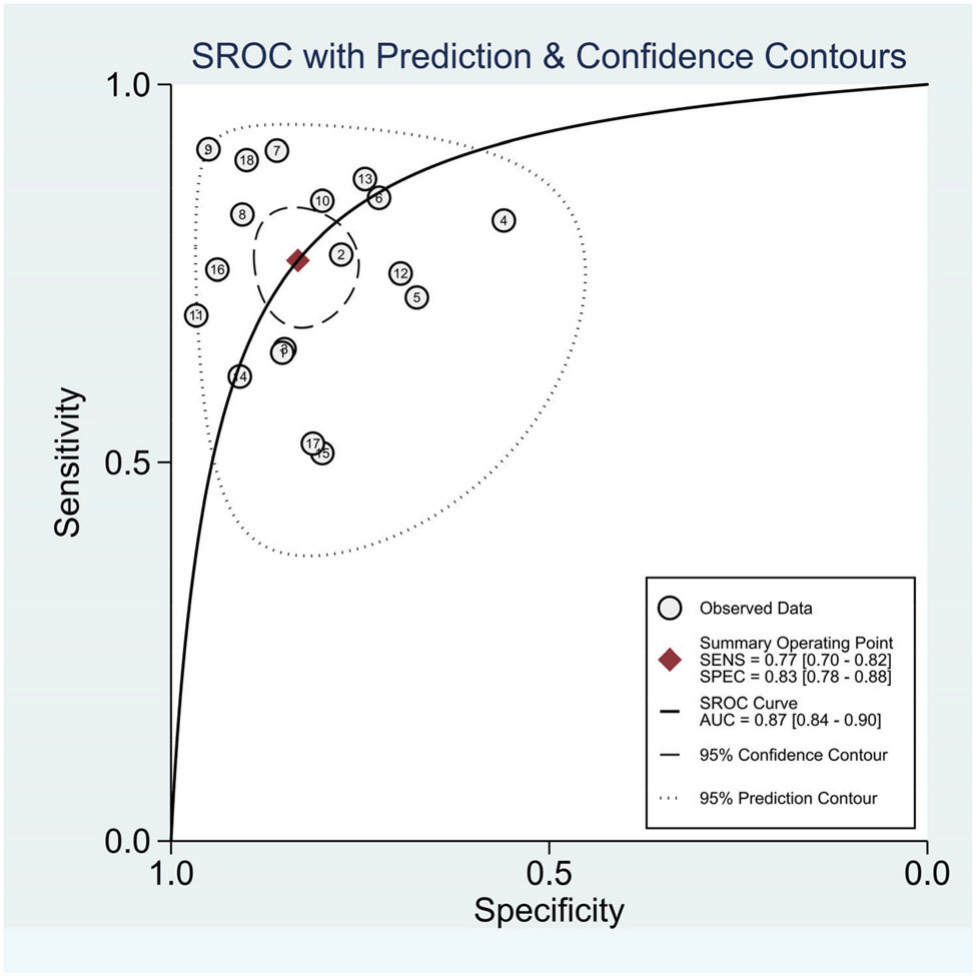
SROC curve for miR-21 in CRC diagnosis.

**Figure 7.**
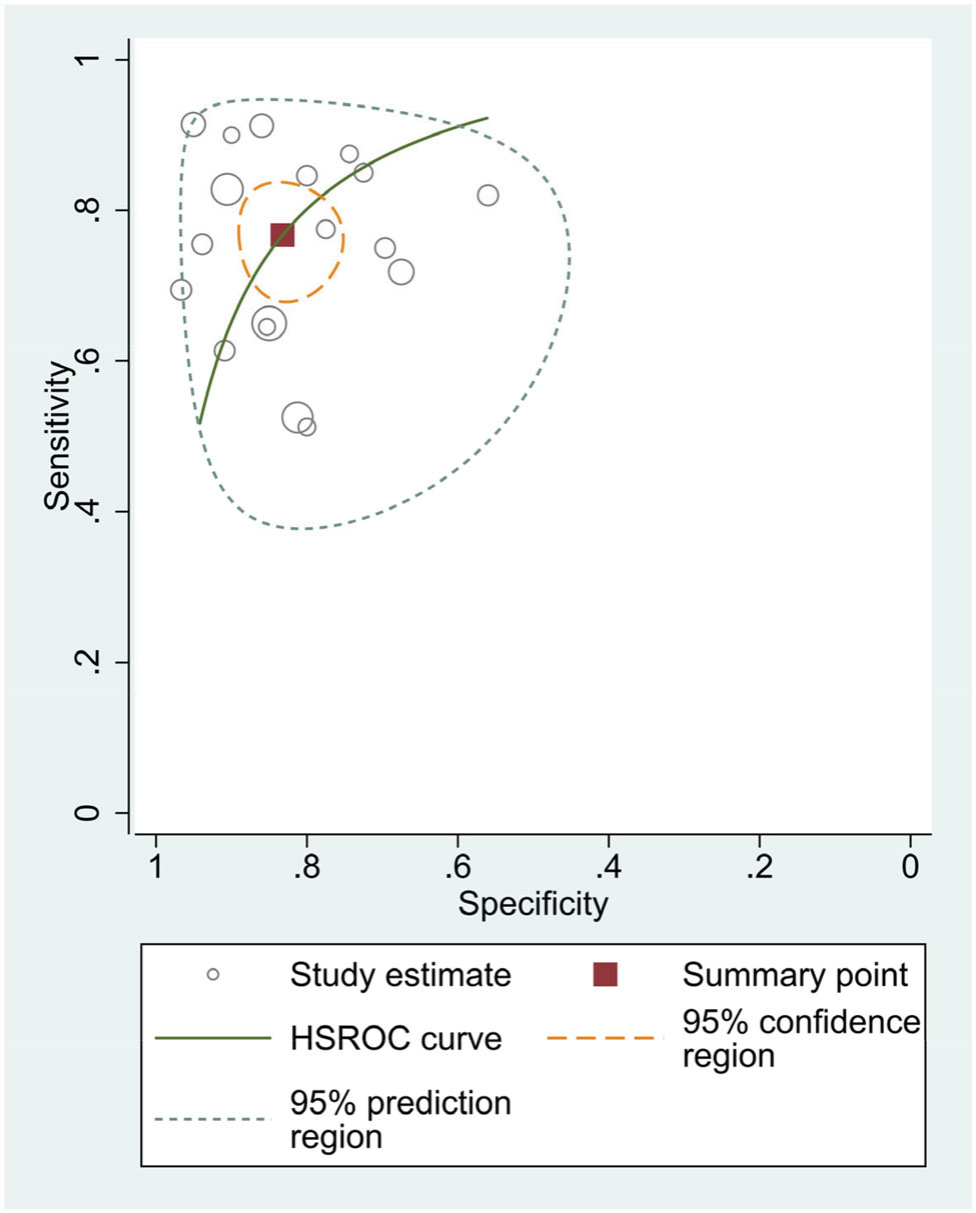
HSROC curve for miR-21 in CRC diagnosis.

The diagnostic odds ratio (DOR) was 16.48 (95% CI 10.09–26.91) (Figure 5). The summary receiver operating characteristic curve (SROC) for the included studies showed the AUC was 0.87(95%CI, 0.84–0.90) (Figure 6), indicating a good diagnostic accuracy. Hierarchical summary receiver operating characteristics (HSROC) curve was shown in Figure 7, and the value of beta was 0.05(95%CI,−0.59–0.70), and the *p*-value was .872, indicating HSROC is symmetrical. The value of Lambda was 2.78 (95%CI, 2.27–3.29), indicating that circulating miR-21 is an accurate diagnostic marker for CRC.

### 3.3. Publication bias

**Figure 8.**
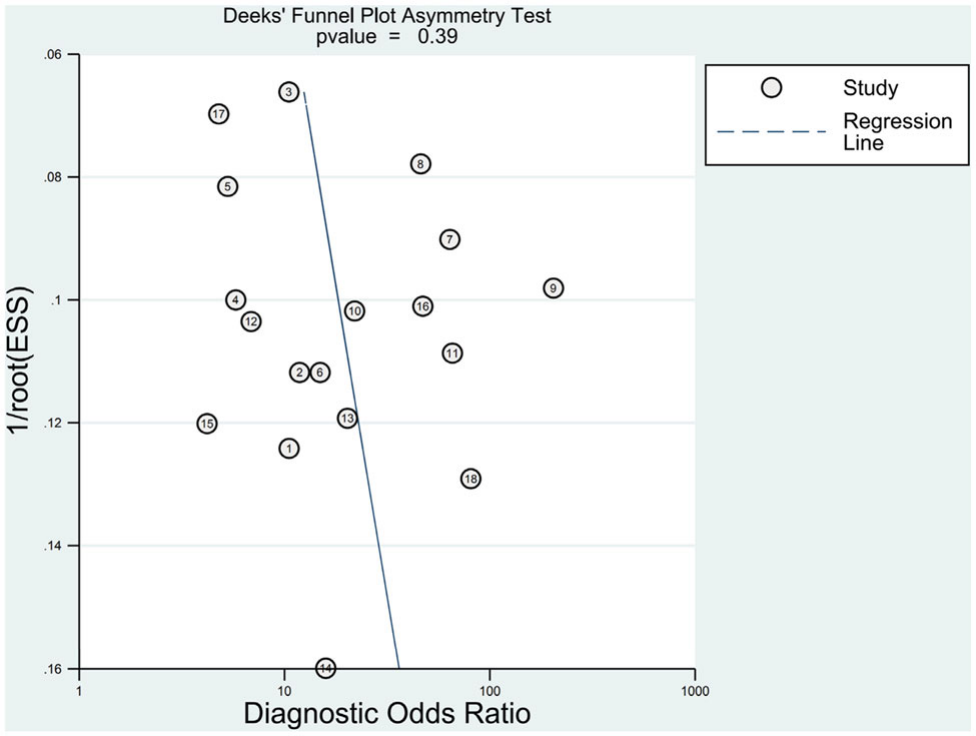
Deeks’ tests for the assessment of publication bias in miR-21 assays.

Deeks' funnel plots were employed to evaluate publication bias in this meta-analysis. As shown in Figure 8, the funnel plot presents symmetry and the *p*-value was .39, indicating there is no publication bias in this meta-analysis.

## 4. Discussion

**Figure 9.**
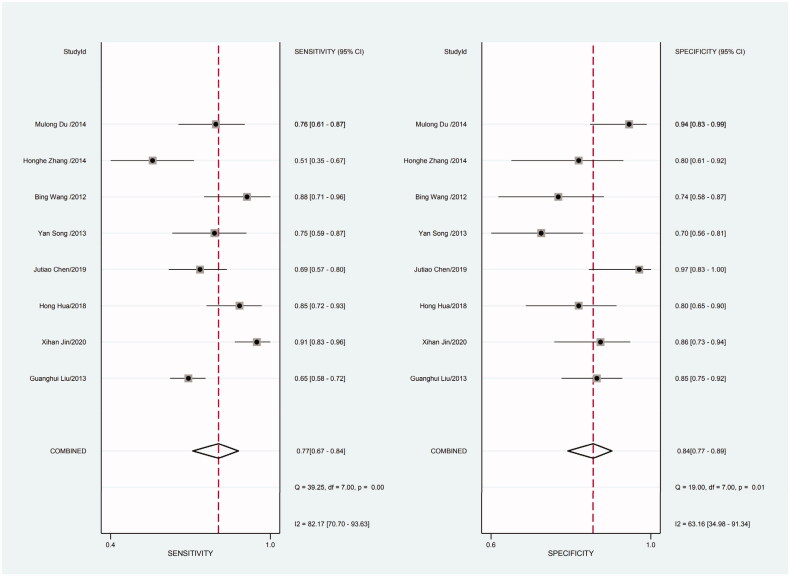
Forest plot of included Chinese studies assessing the sensitivity and specificity of circulating miR-21 in CRC.

**Figure 10.**
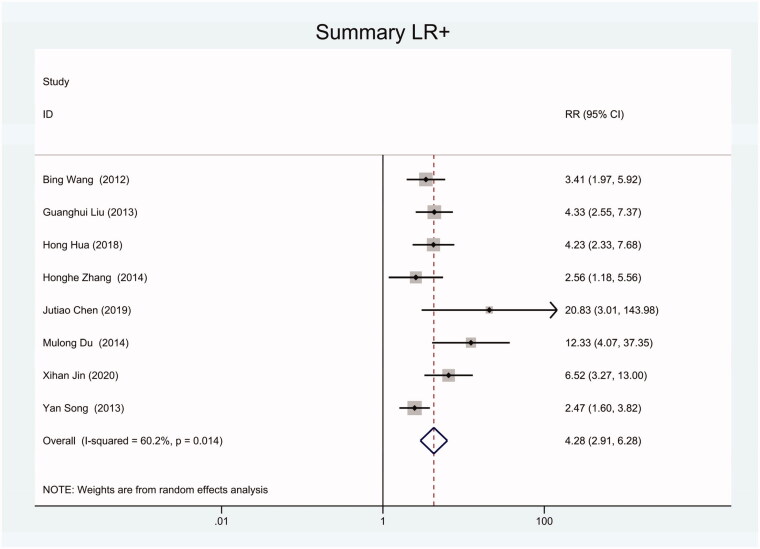
Forest plot of included Chinese studies assessing the PLR of circulating miR-21 in CRC.

**Figure 11.**
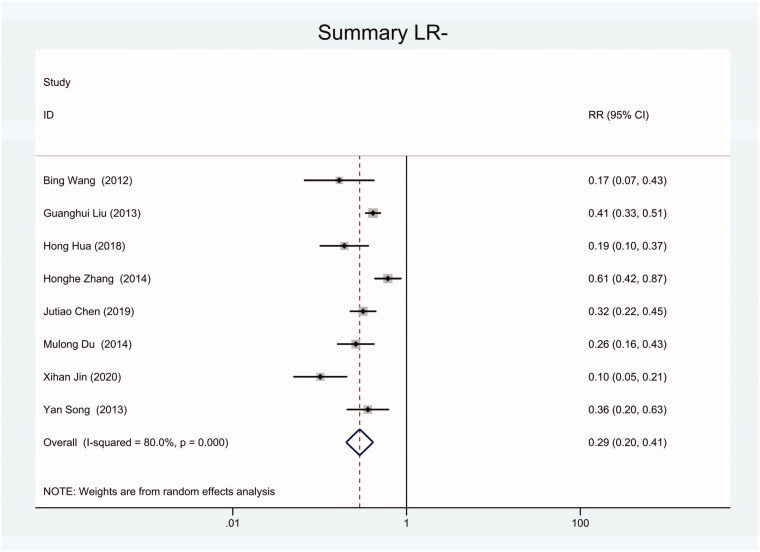
Forest plot of included Chinese studies assessing the NLR of circulating miR-21 in CRC.

**Figure 12.**
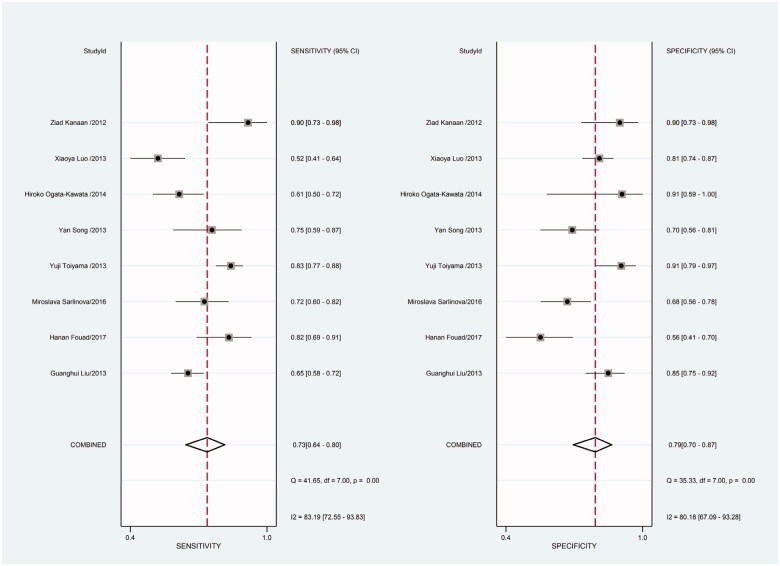
Forest plot of included studies using SYBR-Green qRT-PCR as test method assessing the sensitivity and specificity of circulating miR-21 in CRC.

**Figure 13.**
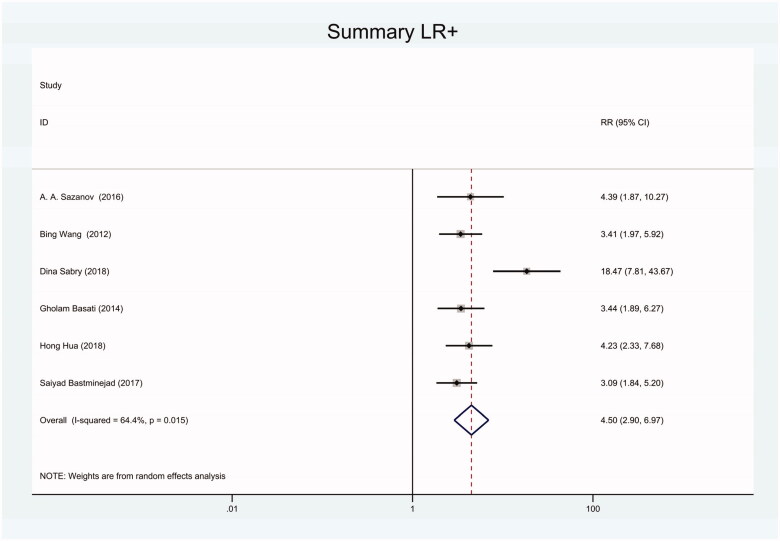
Forest plot of included studies using SYBR-Green qRT-PCR as test method assessing the PLR of circulating miR-21 in CRC.

**Figure 14.**
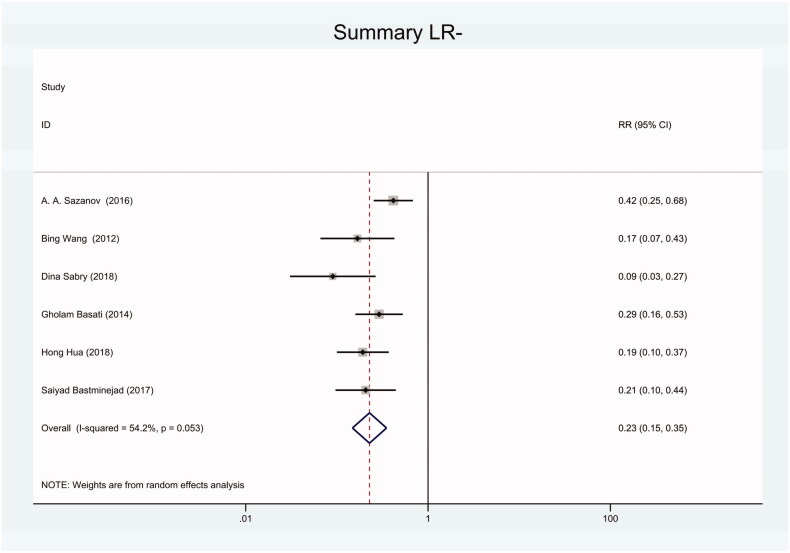
Forest plot of included studies using SYBR-Green qRT-PCR as test method assessing the NLR of circulating miR-21 in CRC.

**Figure 15.**
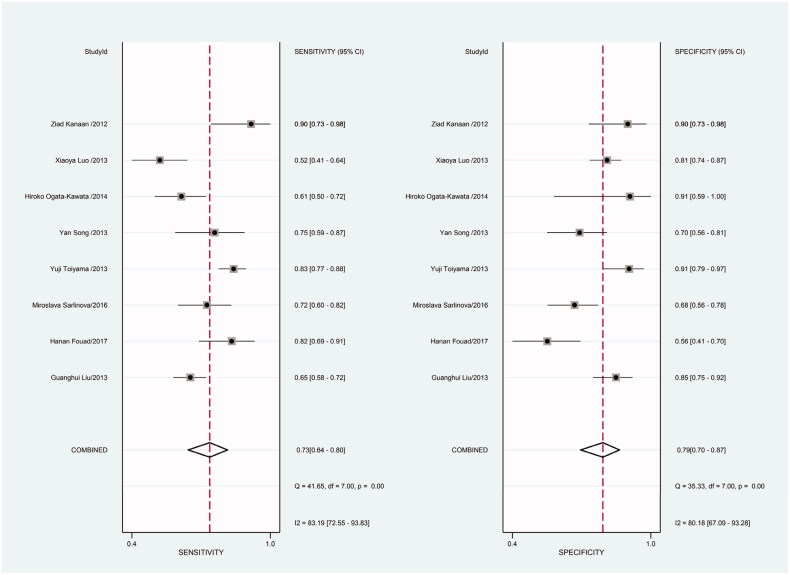
Forest plot of included studies using TaqMan qRT-PCR as test method assessing the sensitivity and specificity of circulating miR-21 in CRC.

**Figure 16.**
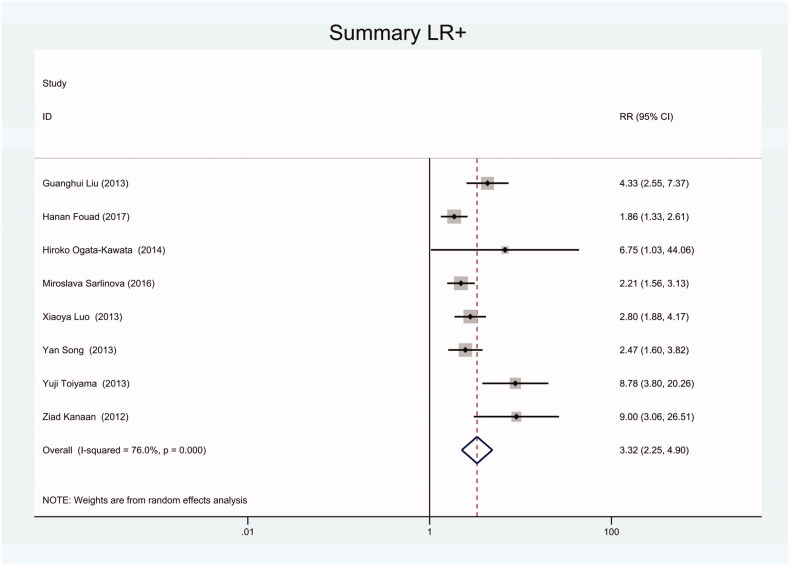
Forest plot of included studies using TaqMan qRT-PCR as test method assessing the PLR of circulating miR-21 in CRC.

**Figure 17.**
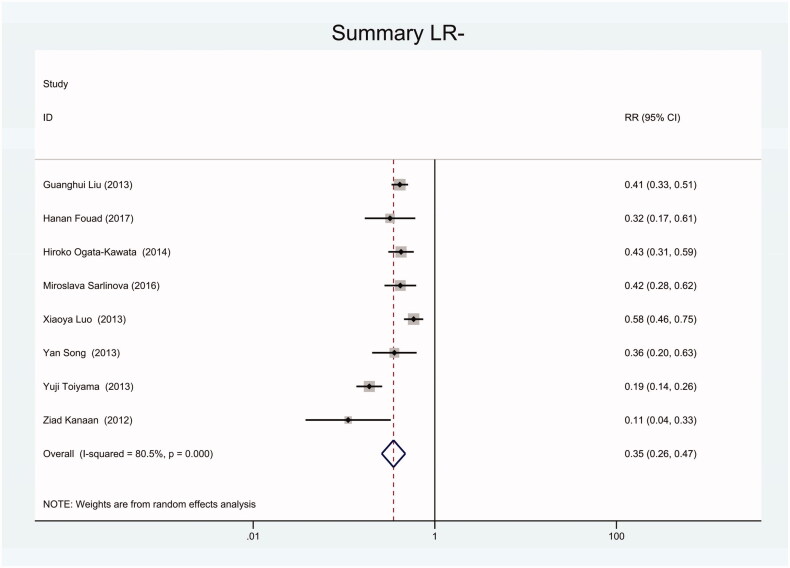
Forest plot of included studies using TaqMan qRT-PCR as test method assessing the NLR of circulating miR-21 in CRC.

**Figure 18.**
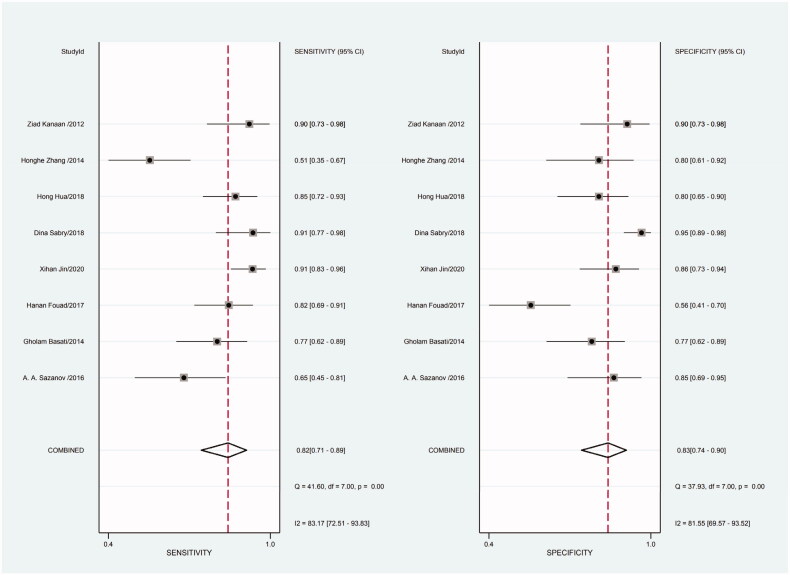
Forest plot of included studies using U6 snRNA as internal reference gene assessing the sensitivity and specificity of circulating miR-21 in CRC.

**Figure 19.**
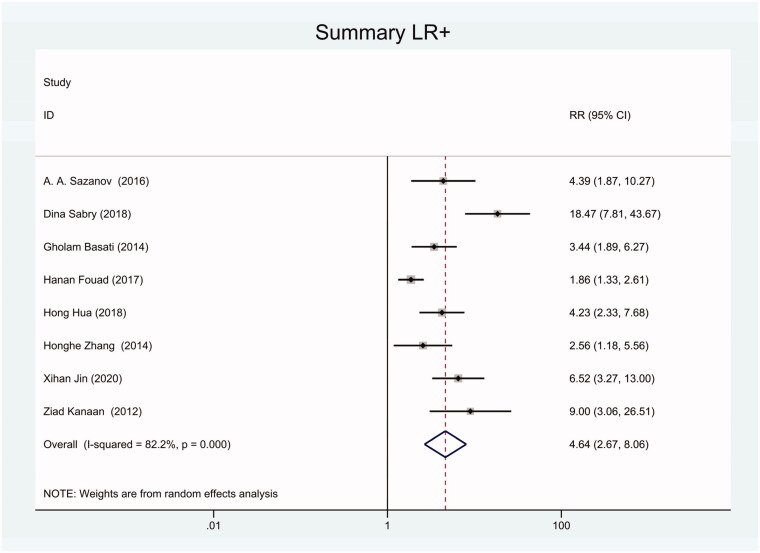
Forest plot of included studies using U6 snRNA as internal reference gene assessing the PLR of circulating miR-21 in CRC.

**Figure 20.**
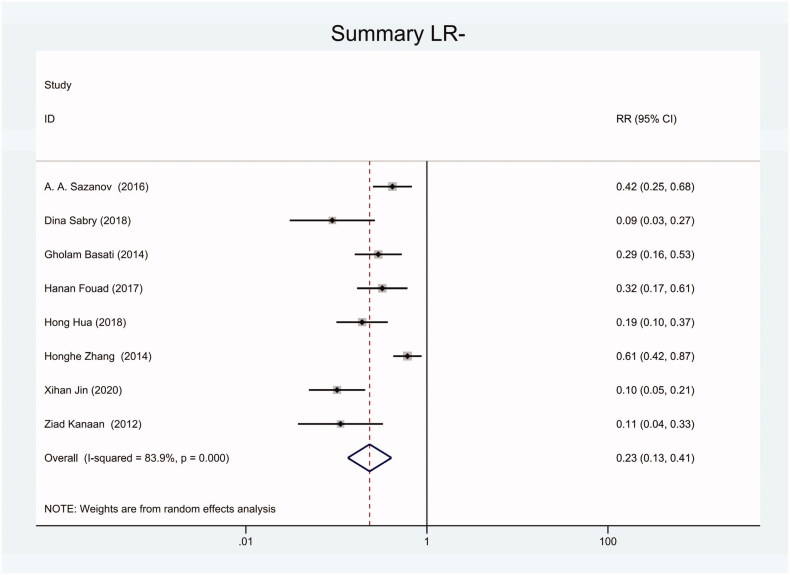
Forest plot of included studies using U6 snRNA as internal reference gene assessing the NLR of circulating miR-21 in CRC.

**Figure 21.**
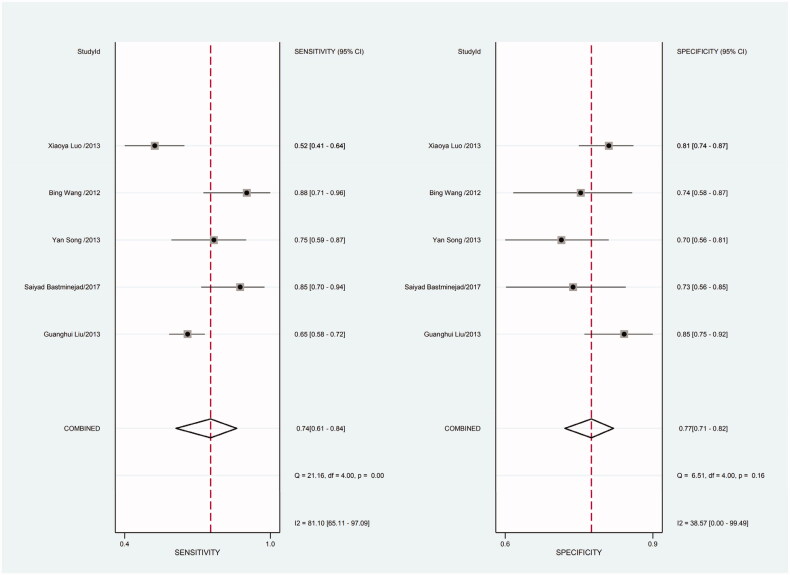
Forest plot of included studies using miR-16 as internal reference gene assessing the sensitivity and specificity of circulating miR-21 in CRC.

**Figure 22.**
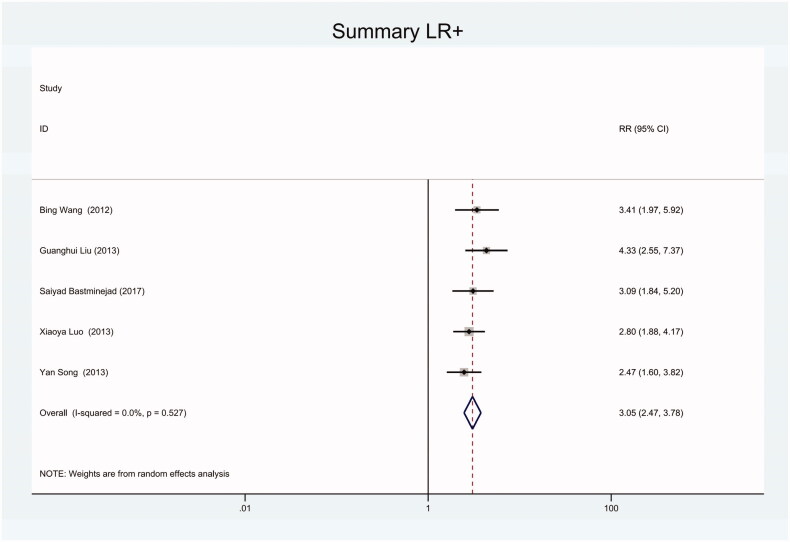
Forest plot of included studies using miR-16 as internal reference gene assessing the PLR of circulating miR-21 in CRC.

**Figure 23.**
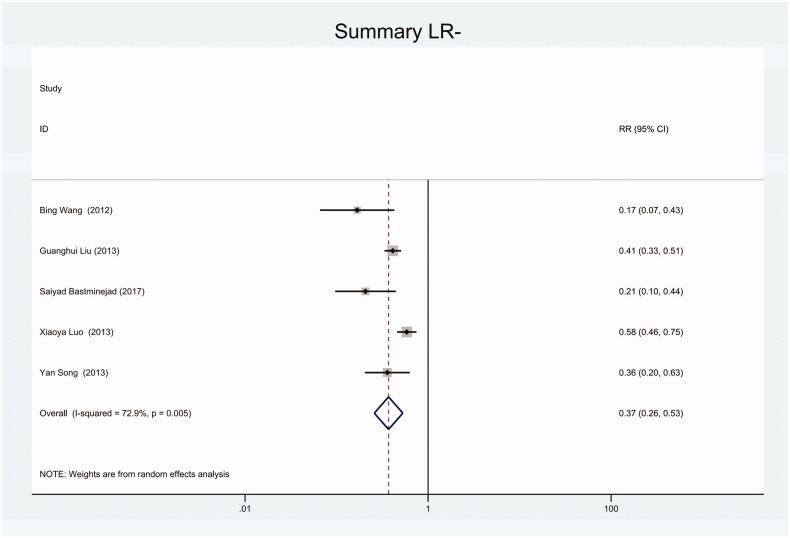
Forest plot of included studies using miR-16 as internal reference gene assessing the NLR of circulating miR-21 in CRC.

As there was heterogeneity in sensitivity and specificity in this meta-analysis, we employed random-effects model. Besides, we also conducted subgroup meta-analysis. There were 8 included studies coming from China, 6 studies using SYBR-Green qRT-PCR as test method, 8 studies using TaqMan qRT-PCR as test method, 8 studies applying U6 snRNA as internal reference gene and 5 studies applying miR-16 as internal reference gene. We conducted subgroup meta-analysis to observe the heterogeneity of each group respectively (Figures 9–23). The results shown that there was no heterogeneity in specificity in group using miR-16 as internal reference gene, and all the other groups had heterogeneity.

To explore the potential source of heterogeneity, we tried to find other factors in heterogeneity. We supposed whether gender and age might be factors in heterogeneity. To confirm our hypothesis, we performed meta regression to investigate the heterogeneity of covariates. Unfortunately, there was no statistically significant heterogeneity about gender and age in this meta-analysis (Table 2). We suspected the tumour stage of the patients might be the factor in heterogeneity, but most included studies in this meta-analysis did not provide details of the tumour stage. Therefore, we cannot analyse the heterogeneity from tumour stage data.

**Table 2.** Possible sources of heterogeneity of covariants in the meta-regression analysis.

	Coef	*p*	95%CI
Age	−0.08	.2	(−0.2;0.05)
Gender	−0.81	.17	(−2.01;0.4)
Country	−0.1	.48	(−0.42;0.21)

Coef: Coefficent.

Several studies have reported the meta-analysis result of circulating miR-21 as diagnostic marker in CRC [15,16], and we read these studies for reference. Compared with these meta-analysis published before, we included more studies to conduct this meta-analysis. Based on QUADAS scores, all included studies had high quality. The included studies were from 8 different countries with 1129 blood specimens of CRC patients and 951 control specimens, which enriched the meta-analysis data of miR-21 as diagnostic marker in CRC published before. Hence, this meta-analysis result was reliable and valuable.

## 5. Conclusion

Our meta-analysis results suggest that circulating miR-21 has a potential diagnostic value with moderate sensitivity and good specificity for CRC. However, more high quality studies about circulating miR-21 diagnostic role should be conducted in future.
